# LncRNA/miRNA/mRNA ceRNA network analysis in spinal cord injury rat with physical exercise therapy

**DOI:** 10.7717/peerj.13783

**Published:** 2022-07-29

**Authors:** Jiahuan Wu, Xiangzhe Li, Qinghua Wang, Sheng Wang, Wenhua He, Qinfeng Wu, Chuanming Dong

**Affiliations:** 1 Suzhou Science & Technology Town Hospital, Gusu School, Nanjing Medical University, Rehabilitation Medical Center, Suzhou, China; 2 Nantong University, Experimental Animal Center, Nantong, China; 3 Medical College of Nantong University, Department of Anatomy, Nantong, China

**Keywords:** Spinal cord injury, Nucleus pulposus, microRNA, Long noncoding RNA, Competing endogenous RNA

## Abstract

Noncoding RNAs have been implicated in the pathophysiology of spinal cord injury (SCI), including cell death, glial scar formation, axonal collapse and demyelination, and inflammation. The evidence suggests that exercise therapy is just as effective as medical treatment in SCI. However, studies of competing endogenous RNA (ceRNA)-mediated regulation mechanisms in the therapy of SCI with exercise are rare. The focus of this research was to investigate the effect of exercise therapy on the expression levels of long noncoding RNA (lncRNA), microRNA (miRNA), and mRNA in rats with SCI. The RNA-seq technology has been used to examine the differentially expressed circRNAs (DECs), lncRNAs (DELs), miRNAs (DEMs), and genes (DEGs) between SCI and exercise therapy rats. The ceRNA network was established using interactions between miRNAs and mRNAs, as well as between miRNAs and lncRNAs/circRNAs. The Database for Annotation, Visualization, and Integrated Discovery was used to anticipate the underlying functions of mRNAs. Our current study identified 76 DELs, 33 DEMs, and 30 DEGs between groups of SCI rats and exercise therapy rats. Subsequently, these newly discovered ceRNA interaction axes could be important targets for the exercise treatment of SCI.

## Introduction

Neurological disorders such as traumatic spinal cord injury (SCI) and brain injury, central nervous system (CNS), stroke, neurodegenerative diseases, and malignancies are affecting an increasing number of people (McDonald & Sadowsky, 2002). Spinal cord injuries are among the most debilitating conditions on this list, as the affected individuals and their families are frequently deprived of attributes that permanently alter their life (Carter, 2014). Traffic accidents are the leading cause of SCI, followed by violent attacks, sports, falls, and industrial traumas (Ahuja et al., 2017; Furlan et al., 2011).

The white and grey matter of the spinal cord contains nerve cell bodies including ascending and descending tracts. As a result, the various locations and extents of SCI can result in variable degrees of disability, ranging from partial sensory or motor loss to full paralysis below the injured area, along with acute and chronic consequences (Haisma et al., 2007; Adriaansen et al., 2013). Currently, the most common treatment interventions for SCI are conservative surgery and medication (Karsy & Hawryluk, 2019; Domurath & Kutzenberger, 2012). However, owing to the loss of nerve tissue, the recovery ability of nerves is limited. For that reason, more research into the treatment and cure for SCI is required to establish more effective techniques for the prevention and treatment of this condition.

Physical activity and exercise are becoming increasingly important in the prevention and treatment of a variety of medical disorders (Sharif et al., 2018; Booth, Roberts & Laye, 2012). There is growing evidence that exercises benefits recovery of neuromuscular function from SCI (Zbogar et al., 2016; Behrman, Ardolino & Harkema, 2017). Physical exercise includig treadmill training is a frequently used approach for restoring the ability to walk after SCI. This extensive exposure of task-specific repetitive training helps promote reorganization of the primary motor cortex (Ungerleider, Doyon & Karni, 2002). Treadmill training significantly improves functional recovery and neural plasticity after incomplete spinal cord injury (Sun et al., 2013). Treadmill training significantly increased the expression of a neurotrophin brain-derived neurotrophic factor (BDNF) in the lumbar motoneurons as compared to non-training (Wang et al., 2015). However, the underlying definite explanation on the therapeutic role of Exercise is still mysterious. Noncoding RNAs, such as circular RNAs (circRNAs), long noncoding RNAs (lncRNAs), and microRNAs (miRNAs/miRs), have recently been discovered to have important roles in a variety of biological processes, including apoptosis and cell proliferation (Lekka & Hall, 2018; Zhao et al., 2020). The miRNAs act by binding to complementary sequences in the 3′-untranslated region (UTR) of their target mRNAs, preventing the transcript from being translated or causing it to be degraded (Achkar, Cambiagno & Manavella, 2016). Binding competitively to miRNAs along with their miRNA response regions, lncRNAs and circRNAs can perform as competing endogenous RNAs (ceRNAs), controlling the miRNA expression levels targeting mRNAs (Panda, 2018). So, the interaction of lncRNA/circRNA-miRNA-mRNA may be considered as a significant mechanism underlying the development and initiation of SCI. Previous research has supported this hypothesis (Peng et al., 2020; Wang et al., 2020). Certain miRNAs (miR-21, miR-486, miR-20) are dysregulated and associated with SCI through regulating inflammation, gliosis, cellular death, or regeneration, in microarray or sequencing investigations (Nieto-Diaz et al., 2014). We predicted that exercise might impact the lncRNA/circRNA-miRNA-mRNA interactions.

The focus of this research was to reveal the lncRNA-miRNA-mRNA ceRNA-mediated regulation pathways in exercise-treated rats as a preliminary approach. This study may provide the influences of such exercise therapies on the injured spinal cord.

## Materials & Methods

### Establishment of spinal cord injury models

We obtained all the adult female Sprague Dawley rats (220–250 g) from the Animal Care Facility of Nantong Medical University (Nantong, China). All animal experiments were performed in accordance with institutional norms and were permitted by Nanjing Medical University’s Animal Care and Use Committee (S20200317-024). All animals were bred and housed (five per cage) in a controlled environment with an ambient temperature of 23 ± 2 °C and free access to water and food was given to animals for a 12-hour light-dark cycle. Rats received a hemisection SCI at the spinal level of Thoracic 10 as described in our previous report. The hemisection surgery was carried out by an expert in the SCI rat model to eliminate the error caused by the incision. Following surgery, an intramuscular injection of the Penicillin 20IU/d (i.m.) was given to the animals for 7 days after the operation. Rats were given bladder care three times each day for two weeks, or until the recovery of bladder control. Baytril (0.06 mg/kg) was injected subcutaneously two times per day for 7 days if there was any sign of infection. Certain criteria were considered for animals being included in or excluded from the experiment. The animal that met any two of the group I criteria ((a) rough coat and unkempt, (b) eyes completely or partially closed for 10 minutes, (c) markedly diminished resistance to being handled (grimace response), (d) markedly decreased movement/lethargy, (e) hunched posture, and (f) distended abdomen), were excluded and euthanized. Euthanasia was performed on mice who met one of the group II criteria ((a) inability to eat or drink, (b) moribund/unresponsive, (c) failure to right itself when placed on its back, (d) dyspnea, or (e) 15% or greater reduction in body weight). The rats were sacrificed two weeks after the second operation and the epicenter of the T10 spinal cord was collected for sequencing and q-PCR analysis. All mice were euthanized humanely, *via* carbon dioxide asphyxiation (all rats were included in the analysis).

### Exercise therapy administration

Animals were given body weight-supported Treadmill Training (TMT) for two weeks starting one week after SCI. The feet of the rats were secured to the pedals and suspended horizontally. The treadmill speed was kept at 6 m/min, and each exercise bout comprised two 20-minute workout intervals per day, five days per week for two weeks. The weight-supported range throughout the training was set at 20%–40% of the rat’s weight, depending on the rat’s functional state. Treatments were administered daily in the same arrangement and at the same time, and cages locations were kept at the same positions throughout the experiment to minimize confounders.

### Sample size for RNAseq studies

The RNA-seq study’s error is caused by two factors: the sequencing’s technical variability , and the biological variability of the specimens being compared within groups. For human samples, this value is frequently 4-1, but for inbred animal lines, it is frequently 1 or less. That is, a 2-fold difference is observed in mean expression between treated and untreated samples, while also seeing a 50% variation in expression within the control or treatment groups. This would correspond to an effect size of 2 and a biological coefficient of variation in group 1 /or both (CV) of 0.5. In general, sequencing depths greater than 5/CV^2^ result in only minor increases in study efficiency and/or power, but adding more samples is always efficient.

Depth is a required argument; any one of the others may be left missing and the function will solve for it. An argument may be a vector, in case a vector of values is returned. If multiple arguments are vectors a matrix or array of results is returned. Values for alpha and power would be 0.05 and 0.9. The effect parameter specifies biological effect that, if it were true, the experimenter would want to be able to detect; commonly used. The statements that A has twice the expression of B has half of A are symmetric, values of 1.5 and 2 will the same result. The estimated CV of expression within group may be the most difficult parameter to choose the in depth discussion recommended values for types of data. Samples sizes in the two groups are equal (*n*2 = *n*) if a second sample is not . Likewise the coefficients of variation in the two groups are assumed to be equal if cv2 is not specified. We show the tables of sample size results at different depths according to each power value through https://git.bioconductor.org/packages/RNASeqPower (Table S1–S3).

### miRNA extraction and sequencing

In total, six samples (three from the SCI group and three from the SCI plus TMT group) were selected for high-throughput RNA sequencing. For each sample, three random rats spinal cord tissues in the same group were mixed together before RNA extraction. A five mm spinal cord was taken from the intact and injured site, the spinal cord segments were rapidly frozen on dry ice following the storage at −80 °C before RNA extraction. After total RNA was extracted by Trizol reagent kit (Invitrogen, Carlsbad, CA, USA), the RNA molecules in a size range of 18–30 nt were enriched by polyacrylamide gel electrophoresis(PAGE). After adding the 3′ adapters, the 36–44 nt RNAs were enriched. Additionally, the 5′ adapters were ligated to the RNAs. The ligation products were reverse transcribed using PCR amplification. The PCR products, which ranged in size from 140 to 160 bp, were enriched to create a cDNA library and sequenced on an Illumina HiSeqTM 2500 (Gene Denovo Biotechnology Co, Guangzhou, China). Results obtained from sequencing contained noise which would affect the following assembly and analysis. Thus, to get pure tags, raw reads were further filtered according to the fastqc software. Then, all purified tags were aligned with GeneBank, Rfan, and Genome. miRNA including existing miRNA, known miRNA, and novel miRNA was identified by software Mireap_v0.2. After tags were annotated as mentioned previously, the annotation results were determined in this priority order: rRNA etc > exist miRNA > exist miRNA edit > known miRNA > repeat > exon > novel miRNA > intron. The tags that cannot be annotated as any of the above molecules were recorded as unann. Correlation analysis of two parallel experiments provides the evaluation of the reliability of experimental results as well as operational stability. The correlation coefficient between two replicas was calculated to evaluate repeatability between samples. The closer the correlation coefficient gets to 1, the better the repeatability between two parallel experiments. We identified miRNAs with a fold change ≥2 and *P* value <0.05 in a comparison as significant DE miRNAs.

### mRNA and LncRNA bioinformatics analysis

Total RNA extraction was performed according to the manufacturer’s procedure using the Trizol reagent kit (Invitrogen, Carlsbad, CA, USA) . The integrity of the RNA was determined using an Agilent 2100 Bioanalyzer (Agilent Technologies, Palo Alto, CA, USA) and validated using RNase free agarose gel electrophoresis. After extracting total RNA, rRNAs were removed to retain mRNAs and ncRNAs. The enriched mRNAs and ncRNAs were fragmented into short fragments by using fragmentation buffer and reverse transcribed into cDNA with random primers. Second-strand cDNA was synthesized by DNA polymerase I, RNase H, dNTP (dUTP instead of dTTP) and buffer. Next, the cDNA fragments were purified with QiaQuick PCR extraction kit (Qiagen, Venlo, the Netherlands), end repaired, poly(A) added, and ligated to Illumina sequencing adapters. Then UNG (Uracil-N-Glycosylase) was used to digest the second-strand cDNA. The digested products were selected on the bases of size by agarose gel electrophoresis, PCR product, and sequenced using Illumina HiSeqTM 4000 (or other platforms) by Gene Denovo Biotechnology Co. (Guangzhou, China). Clean reads were acquired using fastqc software (version 0.18.0) and reads were then mapped to the ribosomal RNA (rRNA) database using the short read alignment tool Bowtie2 (version 2.2.8). After that, the reads that had been mapped to rRNA were deleted. The remaining reads were further used in the assembly and analysis of the transcriptome. An index of the reference genome was built, and paired-end clean reads were mapped to the reference genome using HISAT2 (version 2.1.0). The reconstruction of transcripts was carried out with software Stringtie. To identify the new transcripts, all reconstructed transcripts were aligned to reference genome and were divided into twelve categories using Cuffcompare. Transcripts with one of the class codes “u,i,j,x,c,e,o” were defined as novel transcripts. Then we used the length of the transcript which was longer than 200bp and the exon number was more than 2 to identify reliable novel genes. Two software *i.e.,* CNCI (version 2) and CPC (version 0.9-r2) (http://cpc.cbi.pku.edu.cn/), with default parameters, were used to assess the protein-coding potential of novel transcripts. The intersection of both non protein-coding potential results was chosen as long non-coding RNAs.

Transcripts abundances were quantified by software StringTie is a reference-based approach. For each transcription region, an FPKM (fragment per kilobase of transcript per million mapped reads) value was calculated to quantify its expression abundance and variations. The coding and non-coding RNA transcripts that were differently expressed were investigated, accordingly. DESeq2 was then used to compare the differential expression between two groups (or by edgeR between two samples). The genes/transcripts with the parameter of false discovery rate (FDR) below 0.05 and absolute fold change ≥2 were considered as differentially expressed genes/transcripts.

### Real-time qRT-PCR validation

For expressions quantification, total RNA from spinal cord samples was extracted by using TRIzol reagent (Pufei biological, USA). Extracted RNA was converted into cDNA using a reverse transcription kit (Vazyme, Nanjing, China) as per the instructions of the manufacturer. Internal control for the relative expressions of the gene was set as glyceraldehyde-3-phosphate dehydrogenase gene (GAPDH). Relative levels of gene expression were calculated using the 2-ΔΔ*Ct* method (*n* = 3). The primers used are given in Table 1.

**Table 1:** qRT-PCR primer sequences.

	Forward primer	Reverse primer
BDNF	TGATGCTCAGCAGTCAA	CACTCGCTAATACTGTCAC
TNF-α	CGGAAAGCATGATCCGAGAT	AGACAGAAGAGCGTGGTGGC
IL-1β	TTCAAATCTCACAGCAGCAT	CACGGGCAAGACATAGGTAG
IL-6	AGCCACTGCCTTCCCTAC	TTGCCATTGCACAACTCTT
GAPDH	CCTCCTGCACCACCAACTGCTT	GAGGGGCCATCCACAGTCTTCT

### Constructing the CeRNA network

For evaluation DELs-DEMs interactions the starBase database (version 2.0; https://starbase.sysu.edu.cn/starbase2/) was used (Li et al., 2014), integrating with miRNA-mRNA interactions to create networking of DEL-DEM-DEG ceRNA employing Cytoscape software (version 3.6.1; http://www.cytoscape.org). Similarly, the DECs and DEMs interactions were coupled and then combined with the miRNA-mRNA interactions using Cytoscape software for creating the network of DEC-DEM-DEG ceRNA.

### Functional enrichment and annotation

Gene ontology (GO) analysis is a popular bioinformatics tool for annotating and analyzing transcriptome data. KEGG is a database that helps researchers better understand the biological system’s advanced functions and applications. The data acquired from GO and KEGG pathway database analysis for DEGs were processed by using the Database for Annotation, Visualization, and Integrated Discovery (DAVID, https://david.ncifcrf.gov/). For the genome study, REACTOME (a pathway database) was employed to illustrate the molecular pathway information.

### Statistical analysis

To conduct statistical analysis, we used the GraphPad Prism software. The means ± standard deviations (mean ± SEM) were used to express all of the data. For RT-PCR, three technical replicates were collected from each independent experiment to account for variability in the sample. The significances among multiple groups were determined by One-way Analysis of Variance (ANOVA) followed by Bonferroni post hoc test. *P* value < 0.05 was considered statistically significant.

## Results

### The suppression of the post-SCI inflammatory response by physical exercise therapy

**Figure 1:**
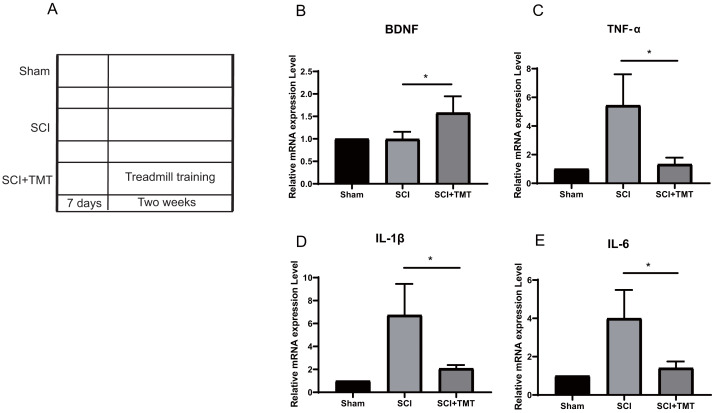
The inhibitory effect of exercise therapy on inflammatory response after SCI. (A) The experimental procedures of the dual rat spinal lesion model and the time course in all rats are shown. TT, treadmill training, treadmill training were performed on rat receiving SCI after 7 days. (B–E) RT-PCR analyses (*n* = 3) of BDNF (*P* = 0.0476), TNF-*α* (*P* = 0.0181), IL-1*β* (*P* = 0.0263), IL-6 (*P* = 0.0265) in the tissue of lesion region were performed two weeks after treadmill training. Data are expressed as mean ± SEM. An asterisk (*) indicates *P* < 0.05 *versus* the SCI treatment groups by one-way ANOVA.

Rats were divided into three groups: (i) Sham; (ii) SCI; (iii) SCI plus TMT (TMT). Firstly, we successfully constructed an SCI model (Fig. S1). In previous studies, SCI-induced microglial activation and successive release of inflammatory factors *i.e.,* tumor necrosis factor (TNF), interferon (INF), and interleukin (IL) have been shown to cause direct death of neurons however induction in vascular endothelial cells for the expression of a range of chemotaxis and cell adhesion molecules (Li et al., 2020a; Li et al., 2020b; Li et al., 2020c; Orr & Gensel, 2018). In our study, within three weeks of post-SCI, the levels of mRNA expressions of IL-6, TNF-α, and IL-1β were increased. Treadmill training was performed after one week of SCI to assess the anti-inflammation potential of physical exercise therapy. Two weeks later, the levels of mRNA of the three pro-inflammatory cytokines at the site of the lesion were examined (Fig. 1A). All three cytokines were markedly suppressed after treatment with physical activity (Figs. 1B–1E).

### Differential expression analysis

**Figure 2:**
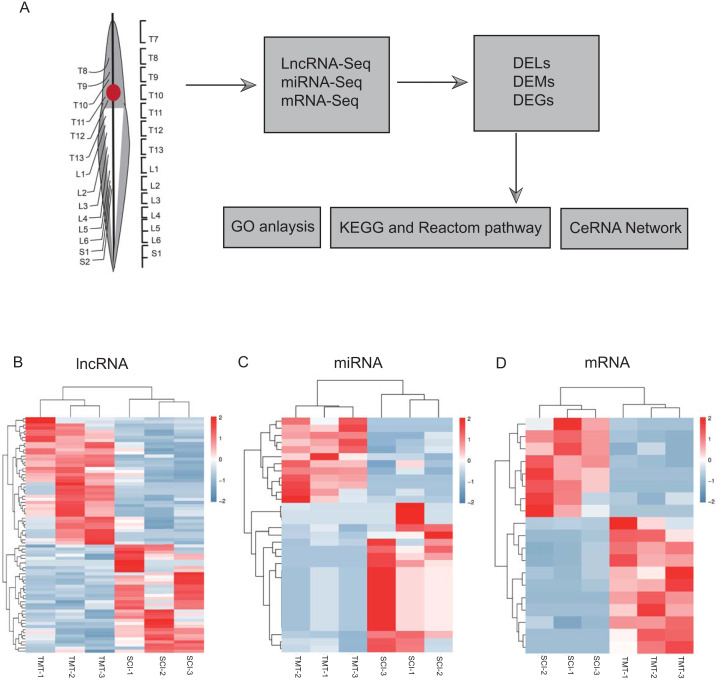
Hierarchical clustering and heat map analysis of differentially expressed, Differentially expressed genes (DEGs) determined by RNA-seq. (A) The workflow of RNA-seq. (B) lncRNA, (C) miRNA and (D) mRNA in spinal cord injury. *N* = 3 rats for the SCI groups (SCI) *versus* SCI plus TMT groups (TMT).

As per the pre-set threshold (FDR value <0.05 and |FC| ≥ 2), we have identified a whole of 76 differential lncRNAs between SCI as well as in control samples, comprising 41 upregulated and 35 downregulated lncRNAs. Whereas 33 differential miRNAs were identified, containing 21 up-regulated and 12 downregulated miRNAs. In the account of mRNAs, 19 differential mRNAs were generated, comprising 11 upregulated and eight downregulated genes. All differential LncRNAs, miRNAs, and mRNAs are presented in Fig. 2 and Tables 2–4.

**Table 2:** LncRNAs identified from spinal cord injury rats and control.

LncRNA	logFC	*P*-value
MSTRG.33.2	10.0812615	0.021022906
ENSRNOT00000076320	8.79224803	0.0107825168758771
MSTRG.11112.11	7.29117069932186	0.035352209
ENSRNOT00000086366	7.129283017	0.0114471563265022
ENSRNOT00000083950	5.824428435	0.0414770168679901
ENSRNOT00000086620	3.432959407	0.0497646771698065
ENSRNOT00000086821	2.669851398	0.0186267039307323
ENSRNOT00000086178	2.427171255	0.00690232969338495
MSTRG.10986.1	2.412693559	0.000160154329580305
ENSRNOT00000086546	1.70160742	0.0152804128373893
MSTRG.4137.1	1.686633919	0.0242778601066535
ENSRNOT00000090814	1.647118977	0.0488143840391992
MSTRG.7062.1	1.51786465	0.00793127349116241
MSTRG.3142.3	1.300233024	0.01383210401143
MSTRG.1588.1	1.134194142	0.000179570539463743
MSTRG.8085.2	1.132755209	0.000168225875882159
MSTRG.19821.1	1.113138975	0.0164053955435965
MSTRG.13512.1	1.087462841	0.0353603256492267
MSTRG.8083.1	1.014592902	0.000319423897631703
MSTRG.2838.2	0.898338025	0.0052426927295388
ENSRNOT00000080918	0.87774425	0.021320614155435
MSTRG.1747.1	0.852631856	0.0177697087990876
MSTRG.17839.2	0.842643672	0.0213202516484291
MSTRG.14369.2	0.812629864	0.0326086535510531
MSTRG.12598.1	0.809435232	0.0415891682203027
ENSRNOT00000076871	0.786495575	0.0326391921614027
MSTRG.19048.1	0.739999654	0.00828216258847988
ENSRNOT00000081763	0.692490965	0.03818921639222
MSTRG.1451.1	0.682359264	0.0394318326751441
MSTRG.14094.1	0.648657176	0.0255286110499475
MSTRG.18736.1	0.647118977	0.017146627923167
MSTRG.17413.3	0.644264249	0.00104354162803263
MSTRG.4152.1	0.619535649	0.00530706474822808
MSTRG.9250.1	0.616196086	0.0229040704386873
MSTRG.1989.1	0.612171348	0.000356634083855439
MSTRG.11374.2	0.607763881	0.0149094189231467
ENSRNOT00000075902	0.604684642	0.0166475071815435
ENSRNOT00000076000	0.604684642	0.0166475071815435
MSTRG.1989.3	0.590292661	0.00215076264719526
MSTRG.16014.1	0.58969731	0.0228027576389219
ENSRNOT00000089399	0.586862034	0.0370607586062419
MSTRG.3891.3	−0.599156382	0.0328769617637782
MSTRG.9743.3	−0.619030082	0.016678145936339
MSTRG.15844.1	−0.666289706	0.00202253984061765
ENSRNOT00000015084	−0.67215706	0.0187497474410401
MSTRG.4710.1	−0.674713027	0.00648103109525139
MSTRG.19470.3	−0.841476083	0.00266923138292542
MSTRG.1989.7	−0.853536791	0.0213306203276205
ENSRNOT00000075969	−0.929873914	0.0347218975818039
ENSRNOT00000076019	−0.959274062	0.00432485597221968
MSTRG.13292.3	−0.979268558	0.000115364004801675
ENSRNOT00000092526	−1.064130337	0.0305839913259358
MSTRG.4415.1	−1.095157233	0.027189419965886
MSTRG.4415.2	−1.175367087	0.0452097190879338
ENSRNOT00000078019	−1.181169759	0.0410096018905549
MSTRG.13261.2	−1.249290905	0.0375332186447193
ENSRNOT00000076087	−1.367855016	0.00169667936735914
MSTRG.11223.1	−1.606495662	0.008217855515345
MSTRG.313.1	−1.635484276	0.0235444573563792
MSTRG.14979.1	−1.705067671	0.0156468198812112
MSTRG.9237.1	−1.719612027	0.0205282663301104
MSTRG.1882.1	−1.883541023	0.00822547540240806
ENSRNOT00000076867	−2.008112646	0.0291130486628121
MSTRG.9743.2	−2.137503524	0.011571659606815
ENSRNOT00000082061	−2.525461489	0.0149733521486662
MSTRG.17976.1	−2.857165222	0.0000002693454398845
MSTRG.12996.1	−2.926386771	0.0205817708129409
MSTRG.19204.13	−3.688158775	0.045320637572825
ENSRNOT00000076212	−5.400879436	0.0426997324624349
MSTRG.9250.2	−7.351675438	0.0384037713653725
ENSRNOT00000076843	−7.437405312	0.0466458729665146
MSTRG.2483.2	−8.196397213	3.60412557930323E−07
MSTRG.18910.6	−8.276124405	0.0277830513033169
ENSRNOT00000085934	−8.667702932	0.0242142523101195
MSTRG.17772.3	−9.299208018	0.0000000155113930641
ENSRNOT00000086103	−9.731319031	0.0387368354233703

**Table 3:** miRNAs identified from spinal cord injury rats and control.

miRNA	LogFC	*P*-value
miR-6783-x	6.635125566	0.007439362
miR-11987-x	6.002852512	0.028075971
miR-4695-y	5.173393982	0.00894195
miR-206-y	4.919975631	0.025565172
novel-m0155-5p	4.91041288	0.019623
miR-8117-y	4.170508397	0.031183564
miR-4443-x	3.003103193	0.000152928
miR-188-x	2.026938811	0.024781164
miR-3969-x	1.977625438	0.041063373
miR-4510-x	1.44232092	0.013940822
novel-m0090-5p	1.250712276	0.049987453
novel-m0094-5p	1.250712276	0.049795445
novel-m0095-5p	1.250712276	0.049794643
novel-m0097-5p	1.250712276	0.04947851
novel-m0099-5p	1.250712276	0.049489535
novel-m0102-5p	1.250712276	0.049638802
novel-m0106-5p	1.250712276	0.049396397
novel-m0109-5p	1.250712276	0.049109307
novel-m0172-3p	1.250712276	0.048731876
rno-miR-1-3p	1.240137998	5.09E−05
rno-miR-206-3p	1.130015606	0.000595429
miR-6325-y	−1.00206838	0.033431201
miR-346-x	−1.540841632	0.032325722
miR-12135-y	−1.711770186	0.00398775
miR-410-5p	−1.975888032	0.010346566
miR-329-x	−2.147890578	0.009034055
miR-1224-y	−2.816357195	0.046707435
miR-487b-5p	−2.827987993	0.030780316
miR-325-x	−3.209453366	0.011362352
miR-421-x	−5.014504078	0.027900932
miR-193b-5p	−5.205939832	0.01268394
miR-381-5p	−5.326009951	0.009075182
novel-m0072-3p	−5.478108949	0.003935518

**Table 4:** mRNAs identified from spinal cord injury rats and control.

mRNA	Gene	LogFC	*P*-value	FDR
ENSRNOG00000000879	Slc9a6	11.67065625	8.88E−27	5.82E−16
ENSRNOG00000050864	LOC100910990	9.366322214	7.13E−20	1.61E−06
ENSRNOG00000010214	Scrn2	8.582455645	1.30E−10	0.044053341
ENSRNOG00000015576	Hsdl1	8.321928095	4.75E−05	0.043050844
ENSRNOG00000009253	Igsf9b	5.257387843	5.45E−05	0.044410478
ENSRNOG00000010018	Clec4a3	4.486012218	4.02E−06	0.004862266
ENSRNOG00000014027	RGD1304728	2.496425826	2.67E−07	0.000543892
ENSRNOG00000014297	Sdc4	2.130703692	6.82E−08	0.000185403
ENSRNOG00000042492	Cop1	1.175309633	1.30E−10	7.08E−07
ENSRNOG00000009329	Nr1d1	1.042908591	4.25E−07	0.00068856
ENSRNOG00000015670	Stx7	0.921466597	4.17E−06	0.004862266
ENSRNOG00000003392	Grsf1	−1.610993791	3.49E−05	0.035612944
ENSRNOG00000019099	AABR07054578.1	−1.800468536	2.29E−07	0.000533957
ENSRNOG00000008249	Brms1l	−1.941017121	4.64E−07	0.00068856
ENSRNOG00000009411	Chn2	−3.732716121	3.77E−07	0.000682447
ENSRNOG00000039494	Aass	−5.240314329	8.73E−06	0.009492712
ENSRNOG00000005975	LOC100362027	−7.93092503	8.88E−27	1.45E−22
ENSRNOG00000006357	Sgip1	−8.965784285	5.03E−08	0.000163971
ENSRNOG00000002070	Mrpl1	−10.14635653	1.06E−06	0.001442195

### GO ontology

**Figure 3:**
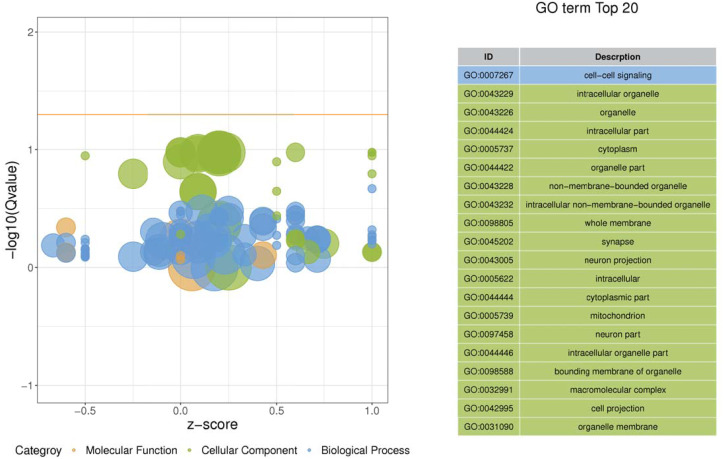
Enriched GO functional categories. GO terms are visualized in semantic similarity-based scatterplots. Bubble color indicates the different categories. Bubble size shows how much the GO term is represented in the GO database (right), Selected top 20 categories are shown on the left.

**Figure 4:**
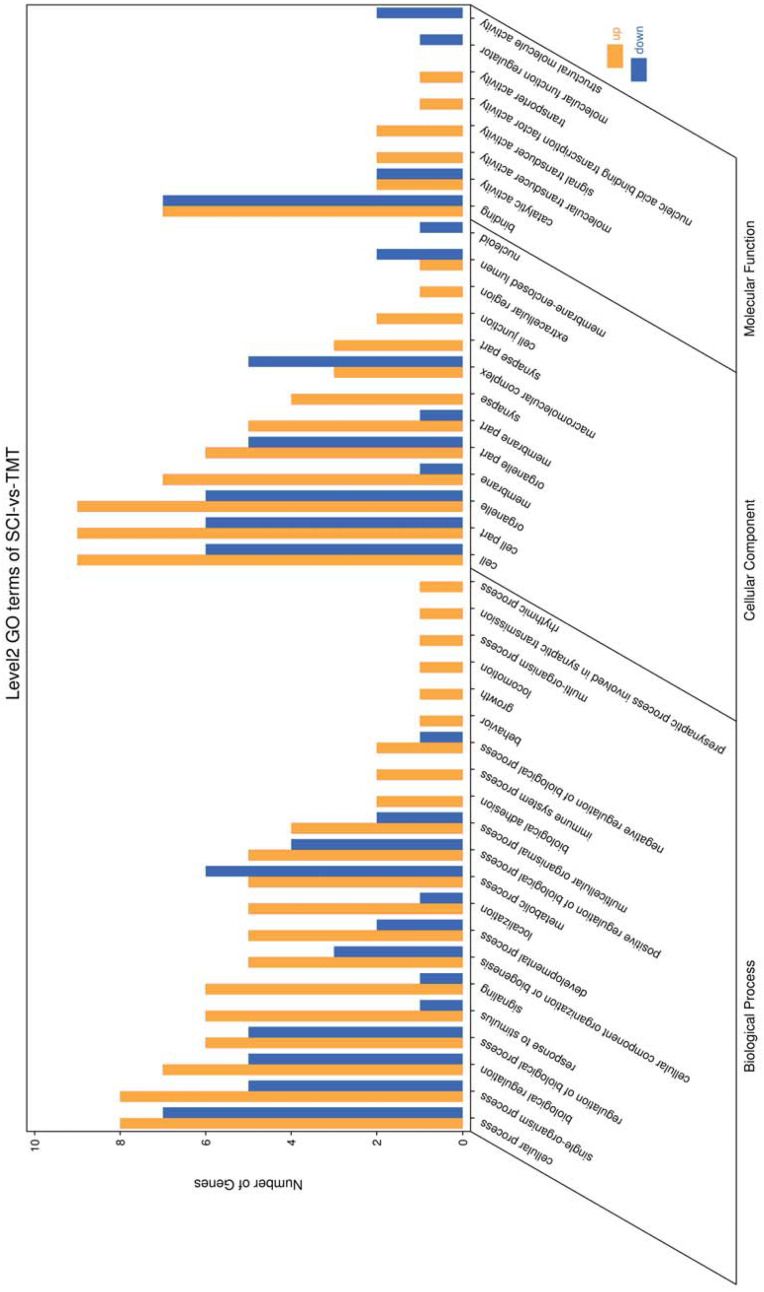
Enriched GO functional categories. GO enrichment analysis shows the numbers of genes in different GO term.

Gene ontology was used to determine which molecular functions and biological processes were overexpressed in DEGs in different analyses employing DAVID 6.8. The top 20 GO terms were shown in Table 4. biological process and 12 cellular components were significantly (*p* < 0.05) identified in “spinal cord injury rats *vs* Control”. Most of these processes were related to cellular components, for example, Intracellular organelle, whole membrane, synapse, neuron projection, mitochondrion, macromolecular complex, organelle membrane (Table 5). The DEGs are mainly involved in eight molecular functions, 13 cellular components, 21 biological processes (Figs. 3–4). In the pathophysiology of SCI, these functions are critical for the control of differently expressed mRNAs.

**Table 5:** Gene ontology analysis of DEGs in spinal cord injury rats.

ID	Descrption	*P*-value	Genes
GO:0007267	Cell–cell signaling	0.008695682	Igsf9b, Nr1d1, Sdc4, Stx7
GO:0043229	Intracellular organelle	0.002179334	Slc9a6, Mrpl1, Grsf1, LOC100362027, LOC100910990
GO:0044424	Intracellular part	0.0153761268449414	Slc9a6, Mrpl1, Grsf1, LOC100362027, Sgip1, Brms1l, Igsf9b, Nr1d1, RGD1304728, Sdc4, Hsdl1, Stx7, Aass, Cop1, LOC100910990
GO:0005737	Cytoplasm	0.012045213	Slc9a6, Mrpl1, Grsf1, LOC100362027, Sgip1, Nr1d1, RGD1304728, Sdc4, Hsdl1, Stx7, Aass, Cop1, LOC100910990
GO:0044422	Organelle part	0.015408745	Slc9a6, Mrpl1, Grsf1, LOC100362027, Sgip1, Brms1l, Nr1d1, RGD1304728, Sdc4, Stx7, Cop1
GO:0043228	Non-membrane-bounded organelle	0.012180255	Mrpl1, Grsf1, LOC100362027, Brms1l, Igsf9b, Nr1d1, RGD1304728, Sdc4
GO:0043232	Intracellular non-membrane-bounded organelle	0.012180255	Mrpl1, Grsf1, LOC100362027, Brms1l, Igsf9b, Nr1d1, RGD1304728, Sdc4
GO:0098805	Whole membrane	0.009392774	Slc9a6, Sgip1, Igsf9b, Sdc4, Stx7
GO:0045202	Synapse	0.007481628	Slc9a6, Igsf9b, Nr1d1, Stx7
GO:0043005	Neuron projection	0.010686377	Slc9a6, Igsf9b, Nr1d1, Stx7
GO:0005622	Intracellular	0.01859359	Slc9a6, Mrpl1, Grsf1, LOC1003620
			27, Sgip1, Brms1l, Igsf9b, Nr1d1, RGD1304728, Sdc4, Hsdl1, Stx7, Aass, Cop1, LOC100910990
GO:0044444	Cytoplasmic part	0.020377852	Slc9a6, Mrpl1, Grsf1, LOC100362027, Sgip1, RGD1304728, Sdc4, Hsdl1, Stx7, Aass, Cop1
GO:0005739	Mitochondrion	0.025294767	Mrpl1, Grsf1, Hsdl1, Aass
GO:0097458	Neuron part	0.029462285	Slc9a6, Igsf9b, Nr1d1, Stx7
GO:0044446	Intracellular organelle part	0.039921176	Slc9a6, Mrpl1, Grsf1, LOC100362027, Sgip1, Brms1l, Nr1d1, RGD1304728, Stx7, Cop1
GO:0098588	Bounding membrane of organelle	0.040810661	Slc9a6, Sgip1, Stx7, Cop1
GO:0032991	Macromolecular complex	0.064570235	Mrpl1, Grsf1, LOC10036202, Sgip1, Brms1l, Nr1d1, Stx7, Cop1
GO:0042995	Cell projection	0.064149656	Slc9a6, Igsf9b, Nr1d1, Stx7
GO:0031090	Organelle membrane	0.103768137	Slc9a6, Sgip1, Stx7, Cop1

**Figure 5:**
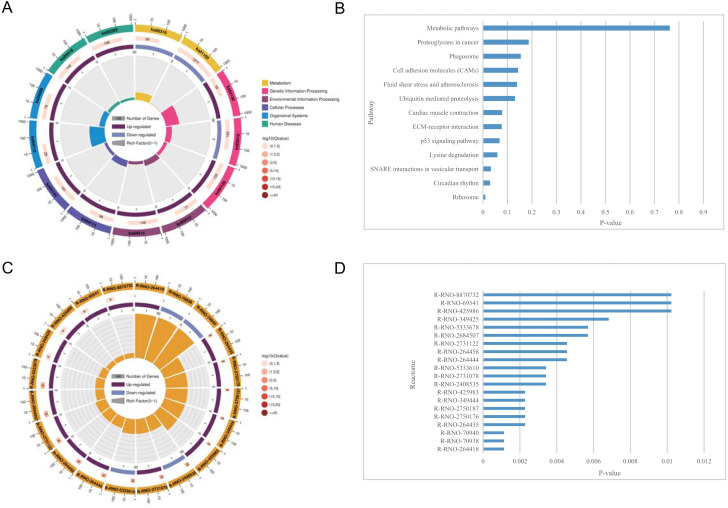
KEGG pathway enrichment and reactome pathway enrichment analysis of DEGs. (A) Circle plot demonstrating the KEGG pathway enriched by the DEGs. Inner plot color corresponds to the rich factor. The second ring displays up and down genes. The third ring displays Q-values. The outer ring displays GO terms. (B) Statistically significant pathways listed and their colors are shown by *P*-value. (C) Circle plot demonstrating the reactome pathway enriched by the DEGs. (D) Top 20 statistically significant pathways listed and their colors are shown by *P*-value.

### KEGG pathway enrichment analysis of the DEGs

The pathways related to DEGs in SCI rats were mostly enriched, according to KEGG analysis. The differentially identified pathways include metabolic pathways, proteoglycans in cancer, phagosomes, cell adhesion molecules, fluid shear stress, and atherosclerosis, ubiquitin mediated proteolysis, cardiac muscle contraction, ECM-receptor interaction, p53 signaling pathway, lysine degradation, SNARE interactions in vesicular transport, circadian rhythm, and ribosome. The fold enrichment of DEGs involved in each pathway was shown by a bar chart(Fig. 5 and Tables 6–7). According to reactome pathway enrichment analyses, PSGs bind proteoglycans, and TGF-beta1 was the most significantly affected phase in SCI. This was supported by the findings of the GO enrichment analysis.

### ceRNA network

**Figure 6:**
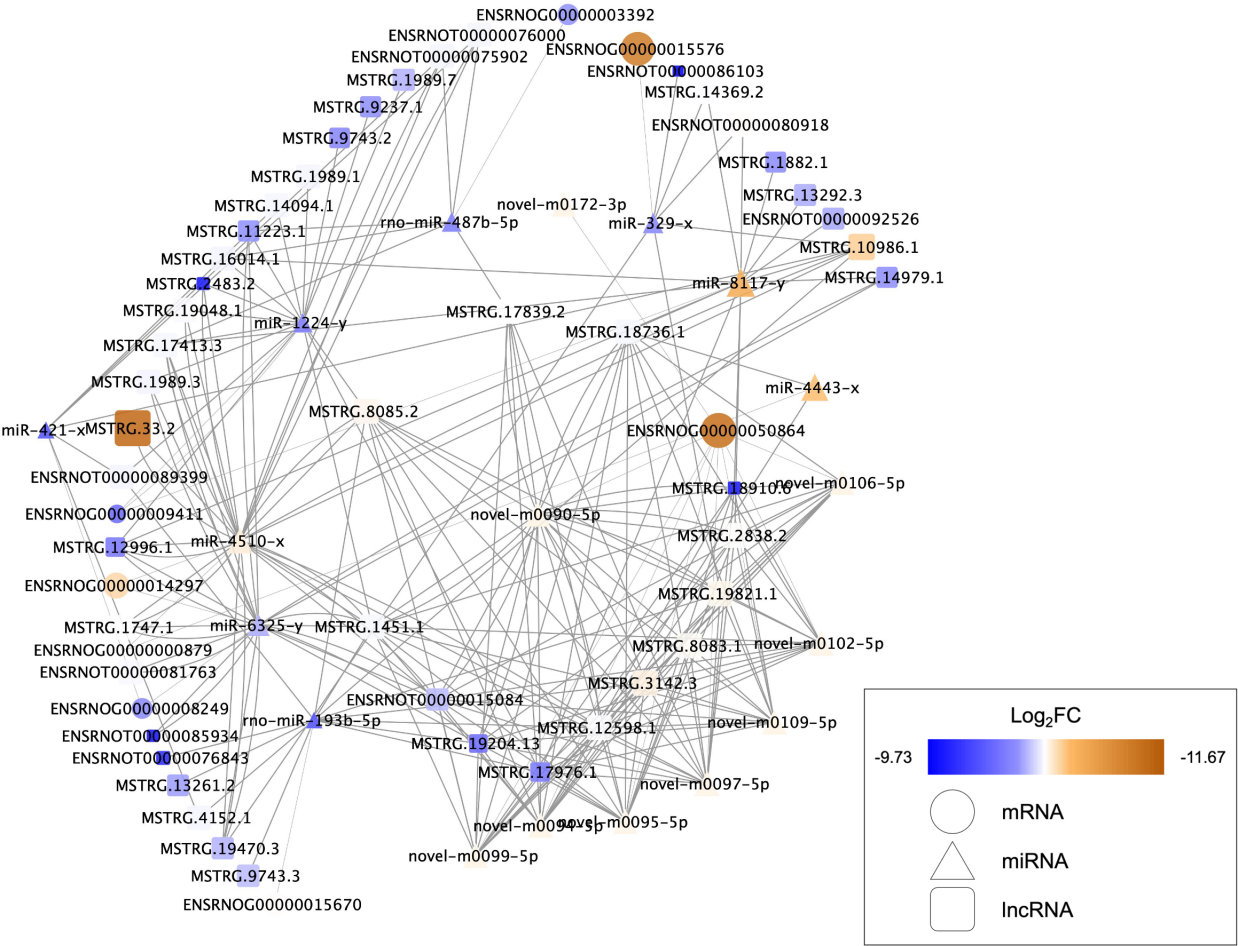
Competing endogenous RNA interaction network of lncRNA-miRNA-mRNA. Blue represents upregulated expression, whereas brown represents downregulated expression. Square nodes represent lncRNAs, triangular nodes represent miRNAs and oval nodes represent mRNAs. Hub genes are indicated by red boxes. FC, fold change; lncRNA, long noncoding RNA; miRNA/miR, microRNA.

Nine DEMs were projected to control 45 DELs using the starBase database, and this information was applied for the development of the lncRNA-miRNA-mRNA ceRNA network, which was then interconnected to the miRNA-mRNA network. In this network, there were 73 nodes (20 DEMs; 45 DELs; 8 DEGs), and 246 interactions (21 DEL-DEM and 225 DEM-DEG interactions) (Fig. 6). Particularly, endosome-related signaling pathways were considerably enriched in the genes functional analysis in the network of lncRNA-associated ceRNA (Table 8). The detail of ceRNA network show in Table S4.

## Discussion

In current findings, we investigated that the anti-inflammatory effects of physical exercise therapy on the SCI rats, indicating that physical exercise may play the endogenous protection to spinal cord injury in the rats. In addition, the induction of neurotrophins with exercise, especially BDNF, was also confirmed by our data. Physical exercise is an active area of research that is primarily focused on promoting regeneration after disease or injury. Therefore, our research may shed light on the potential of exercise as a therapeutic intervention for SCI. Next, we demonstrated that the mechanism of physical exercise-induced endogenous protection involves anti-inflammatory and regeneration activities.

Recently, genome-wide association studies provide a new perspective on the pathophysiology of diseases at the molecular level (Ma et al., 2020). Noncoding RNAs (ncRNAs) are becoming more widely recognized as gene regulators (Kopp & Mendell, 2018). According to Zhu et al. (2019) these newly discovered ceRNA interaction axes could be an important targets for treating intervertebral disc degeneration, including Metastasis-associated lung adenocarcinoma transcript 1 (MALAT1)/hsa_circRNA_102348-hsa-miR-185-5p-TGFB1/FOS,MALAT1-hsa-miR-155-5p-HIF1A, hsa_circRNA_102399-hsa-miR-302a-3p-HIF1A, MALAT1-hsa-miR-519d-3p-MAPK1, and hsa_circRNA_100086-hsa-miR-509-3p-MAPK1 ceRNA axes. After rigorous selection, Ma et al. (2020) found that the lncRNA-associated networks of ceRNA in the AD mouse model were revealed to be primarily engaged in memory (Akap5), synaptic plasticity, and regulation of amyloid-β (Aβ)-induced neuro-inflammation (Klf4). The ncRNAs have a critical role in the physiology and pathophysiology of SCI. They are also well-known as a promising candidate for disease biomarkers and therapies (Gong, Liu & Shen, 2021). In this study, RNA-seq was employed for systematically analyzing the profiles of mRNA, lncRNA, and miRNA in SCI rats after physical exercise therapy at two weeks post-SCI. In the current study, the SCI rat model was used for T10 lateral hemisection and exercise treatment was employed for 14 days after SCI operation. Finally, the samples of T10 spinal cord segments were collected for RNA-seq.

The genes in the DEGs were analyzed using GO enrichment and KEGG analysis and we recognized not only several enriched terms involved in cell part including intracellular part, organelle part, cytoplasm, whole membrane, the bounding membrane of organelle, but also various pathways relevant to neuronal rewiring including synapse, neuron projection, neuron part. According to reports, exercise promotes neuronal plasticity and stimulates neurogenesis in the adult CNS (Wu et al., 2016; Horowitz et al., 2020). KEGG analysis illuminates the key pathways including metabolic pathways and the p53 signaling pathway, accordingly. Exercise regulates the expression of a multitude of genes in the CNS, including neutrotrophins and genes involved in neuronal plasticity, the immune response, and cell death (Jang et al., 2019; Dupont-Versteegden et al., 2004). As a result, some modulatory molecules acquired from the spinal cord may be responsible for the anti-inflammatory effects of exercise therapy and increased BDNF expression shown in our study. After a traumatic injury, we detected significant changes in the expression of associated ncRNAs and mRNAs in spinal cord tissue, and we projected the structure and possible function of the differentially expressed ncRNAs and mRNAs regulation network. In general, lncRNA and miRNA molecules can have a role in SCI regulation. The LncRNAs and protein-coding mRNAs both act as ceRNAs and super-sponges controlling the expression of miRNA. As a result, we applied miRanda to predict interactions between miRNA–mRNA and miRNA–lncRNA and established triple networks among DElncRNA–DEmiRNA–DEmRNA for SCI in rats receiving physical exercise therapy. The identified networks of lncRNA-associated ceRNA may help to understand further the effects of exercise on SCI and develop new treatments for the disease.

**Table 6:** KEGG pathway of DEGs.

Pathway	*P*-value	Genes
Ribosome	0.01025381	Mrpl1, LOC100362027
Circadian rhythm	0.02951816	Nr1d1
SNARE interactions in vesicular transport	0.03252779	Stx7
Lysine degradation	0.05924911	Aass
p53 signaling pathway	0.06801154	Cop1
ECM-receptor interaction	0.07670248	Sdc4
Cardiac muscle contraction	0.07862414	Slc9a6
Ubiquitin mediated proteolysis	0.1310292	Cop1
Fluid shear stress and atherosclerosis	0.1392034	Sdc4
Cell adhesion molecules (CAMs)	0.1437155	Sdc4
Phagosome	0.1544602	Stx7
Proteoglycans in cancer	0.1868507	Sdc4
Metabolic pathways	0.7628114	Aass

**Table 7:** Reactome pathway of DEGs.

Reactome	Reactome_Name	*P*-value	Genes
R-RNO-264418	Translocation of COP1 from the nucleus to the cytoplasm	0.001140487	Cop1
R-RNO-70938	lysine + alpha-ketoglutarate + NADPH+ H+ = > saccharopine + NADP+ + H2O	0.001140487	Aass
R-RNO-70940	saccharopine + NAD+ + H2O = > alpha-aminoadipic semialdehyde + glutamate + NADH+ H+	0.037331594	Aass
R-RNO-264435	Dissociation of the COP1-p53 complex	0.002279792	Cop1
R-RNO-2750176	Syndecan-4 binds Actn1	0.002279792	Sdc4
R-RNO-2750187	Syndecan-4:PI(4,5)P2 binds PKC alpha:DAG	0.002279792	Sdc4
R-RNO-349444	Phosphorylation of COP1 at Ser-387 by ATM	0.037331594	Cop1
R-RNO-425983	SLC9A6,7 exchange Na+ for H+ across the early endosome membrane	0.037331594	Slc9a6
R-RNO-2408535	Sec-tRNA(Sec):Eefsec:GTP binds to Rpl30	0.040704263	LOC100362027
R-RNO-2731078	Syndecans 2, (4) bind TGFB1	0.040704263	Sdc4
R-RNO-5333610	Rpl30:Met-tRNAi:mRNA:Secisbp2:Sec-tRNA(Sec):Eefsec:GTP is hydrolysed to Rpl30:Met	0.040704263	LOC100362027
R-RNO-264444	Autoubiquitination of phospho-COP1(Ser-387 )	0.042620458	Cop1
R-RNO-264458	Proteasome mediated degradation of COP1	0.042620458	Cop1
R-RNO-2731122	Syndecans 1, 2 & 4 bind VTN	0.042620458	Sdc4
R-RNO-2684507	Syndecans 1, 2, 4, (3) bind FGF2	0.046591965	Sdc4
R-RNO-5333678	CPNEs bind PL	0.046591965	LOC100910990
R-RNO-349425	Autodegradation of the E3 ubiquitin ligase COP1	0.006825207	Cop1
R-RNO-425986	Sodium/Proton exchangers	0.010221905	Slc9a6
R-RNO-69541	Stabilization of p53	0.010221905	Cop1
R-RNO-8870732	PSGs bind proteoglycans and TGF-beta1	0.010221905	Sdc4

**Table 8:** Gene ontology analysis of ceRNA.

Enrichment	Count	*P*-value	Genes
Plasma membrane	4	1.7E−1	Stx7, SLC9A6, Sdc4, Cop1
Membrane	5	2.0E−1	Stx7, SLC9A6, Sdc4, Cop1, LOC100362027
Transmembrane helix	3	6.6E−1	Stx7, SLC9A6, Sdc4
Transmembrane	3	6.7E−1	Stx7, SLC9A6, Sdc4
Integral component of membrane	3	6.9E−1	Stx7, SLC9A6, Sdc4

The data analysis showed different lncRNA-associated ceRNA networks taking part in molecular pathways that may improve recovery in the SCI population through exercise. Syntaxin-7(Stx7) is a syntaxin family membrane receptor involved in vesicle transport (Wang, Frelin & Pevsner, 1997). Syntaxin is involved in vesicle docking and fusion, both of which are required for neurotransmitter release (Hammarlund et al., 2007; Li et al., 2020a; Li et al., 2020b; Li et al., 2020c). Ying et al. (2012) reported that spinal cord can decrease the levels of Syntaxin-3 (Stx3) after traumatic brain injury, which affects membrane homeostasis. The majority of spinal cord vulnerability investigation has concentrated on the effects of syntaxins such as Stx3 and Stx1 (Matsuzawa et al., 2014; Holly et al., 2012). However, there has been little research into the effects of Stx7 on the spinal cord. The ceRNAs and the gene Stx7 are part of one of these networks, including LNC ENSRNOT00000089399, LNC MSTRG.13261.2, LNC MSTRG.1451.1, LNC MSTRG.12598.1, LNC MSTRG.19204.13, LNC ENSRNOT00000076843, LNC MSTRG.17976.1, LNC MSTRG.10986.1, LNC MSTRG.8085.2, LNC MSTRG.4152.1, LNC MSTRG.3142.3, LNC MSTRG.19821.1, LNC MSTRG.9743.3. These ceRNAs show the ability for binding miR-193b-5p, targeting the Stx7. NHE6, an endosomal sodium-hydrogen exchanger known to be important in the regulation of endosomal pH, is encoded by Solute carrier family 9 member A6 (SLC9A6) (Yamashiro et al., 1984; Xinhan et al., 2011). Defects in the SLC9A6/NHE6 transporter have been linked to changes in somatosensory functions, according to a recent study (Sikora et al., 2016; Petitjean et al., 2020). Defects in the spinal cord, the nociceptor level, or supraspinal locations can cause sensory impairments. In the dorsal and ventral horns, as well as along the pericentral canal, NHE6/Y KO animals showed enhanced microglial and astrocytic immunoreactivity, indicating that exercise training may increase the levels of SLC9A6/NHE6 to decrease the sensory impairments after SCI. The current study found that exercise training is effective in alleviating the neuropathic pain caused by partial SCI in rats (Li et al., 2020a; Li et al., 2020b; Li et al., 2020c). In this study, it was discovered that SLC9A6 was elevated. MiR-4443, the same as Stx7, has been demonstrated to influence SLC9A6 expression. Furthermore, MiR-4443 could also be sponged by LNC MSTRG.18736.1 and MSTRG.12598.1. Syndecans-4 (SDC4) is a member of the Syndecans (SDCs) family of transmembrane heparan sulphate proteoglycans located on the cell surface (Leblanc et al., 2018). Through their glycosaminoglycan chains, SDCs have been stated for interacting with growth factors and extracellular matrix molecules. The effects of SDC4 are achieved as an independent receptor for the platelet-derived growth factors (PDGFs), fibroblast growth factor receptors (FGFR1–FGFR4), vascular endothelial growth factors (VEGFs), as well as fibroblast growth factors (FGFs) (Elfenbein & Simons, 2013). However, at present, no study reports the relationship between the SDC4 and BDNF yet. We suspected that SDC4 plays a significant role in the regulation of BDNF. The results of this investigation revealed that miR-6325 may be involved in the regulation of SDC4 expression, whereas MSTRG.17413.3, MSTRG.1989.3, MSTRG.19470.3, MSTRG.11223.1, MSTRG.14979.1, MSTRG.1747.1, ENSRNOT00000015084, MSTRG.12996.1, ENSRNOT00000085934, ENSRNOT00000081763, MSTRG.1451.1, and MSTRG.19204.13 could interact with miR-6325 as a ceRNA.

## Conclusions

In conclusion, the profiles of lncRNA-associated ceRNA of SCI and physical exercise therapy in rats were explained. The regulatory roles of exercise-induced alterations in gene expression and the present understanding of ceRNA biology have been improved by our findings. We identified Slc9a6, Sdc4, Stx7 as crucial genes in SCI rats with physical exercise therapy, which leads to the hypothesis that exercise promotes efficient signaling and neuronal plasticity in the spinal cord.

## Supplemental Information

10.7717/peerj.13783/supp-1 Figure S1 Hemisection SCI at the spinal level of Thoracic 10 (A) Exposed spinal cord following a dorsal laminectomy procedure. (B)Spinal cord injury tissue after 4% paraformaldehyde (PFA) perfused

10.7717/peerj.13783/supp-2 Table S1 Sample size in Group 1 (Sham-1/SCI-1/SCI plus TMT-1) for RNAseq study Given data show the power value results at 10–100 depths according to the coefficient of variation of counts within each of the two groups.

10.7717/peerj.13783/supp-3 Table S2 Sample size in Group 1 (Sham-2/SCI-2/SCI plus TMT-2) for RNAseq study Given data show the power value results at 10–100 depths according to the coefficient of variation of counts within each of the two groups.

10.7717/peerj.13783/supp-4 Table S3 Sample size in Group 1 (Sham-3/SCI-3/SCI plus TMT-3) for RNAseq study Given data show the power value results at 10–100 depths according to the coefficient of variation of counts within each of the two groups.

10.7717/peerj.13783/supp-5 Table S4 lncRNA/miRNA/mRNA ceRNA network

10.7717/peerj.13783/supp-6 Supplemental Information 1 BDNF mRNA raw data

10.7717/peerj.13783/supp-7 Supplemental Information 2 TNF-*α*, IL-6, IL-1*β* mRNA raw data

10.7717/peerj.13783/supp-8 Supplemental Information 3 Author Checklist

## Additional Information and Declarations

### Data Availability

The following information was supplied regarding data availability: The data is available at NCBI SRA: SRR17599934, SRR17599935, SRR17599936, SRR17599937, SRR17599938, SRR17599939.
